# Appraisal of amyloidosis imaging practices in the Middle East/North Africa (PYP-MENA)

**DOI:** 10.1093/ehjimp/qyad025

**Published:** 2024-01-16

**Authors:** Firas Al Badarin, Masoud Garashi, Ahmed Aljizeeri, Ramzi Tabbalat, Adel Allam, Salah Eddine Bouyoucef, Ammar Chauhdary

**Affiliations:** Heart and Vascular Institute, Cleveland Clinic Abu Dhabi, Abu Dhabi, United Arab Emirates; Lerner College of Medicine, Case Western Reserve University, Cleveland, OH, USA; Nuclear Medicine Department, Chest Diseases Hospital, Kuwait City, Kuwait; King Abdulaziz Cardiac Center, Ministry of National Guard, Health Affairs, Riyadh, Kingdom of Saudi Arabia; National Amyloidosis Center, Abdali Hospital, Amman, Jordan; Department of Cardiology, Al Azhar University, Cairo, Egypt; Department of Nuclear Medicine, CHU Bab El Oued, Algiers, Algeria; Department of Cardiology, King Faisal Specialist Hospital and Research Center, Jeddah, Kingdom of Saudi Arabia

**Keywords:** amyloidosis, bone-avid tracers, SPECT

## Abstract

**Aims:**

Whereas recommendations to optimize performance and yield of cardiac scintigraphy studies with bone-seeking tracers have been published, little is known about real-world adherence to these best practices, especially outside North America and Europe. Accordingly, we described imaging practices with this modality in a sample of nuclear laboratories in the Middle East/North Africa (MENA) region.

**Methods and results:**

Laboratories performing radionuclide imaging for cardiac amyloidosis in the MENA region were invited to participate in this study to describe installed camera systems, type and dose of bone-avid tracers used, imaging protocols, and criteria used for study interpretation. Out of 19 invited sites, 10 completed the survey (70% government-run; 90% accredited), sites have been involved with amyloid imaging for a median of 49 months (interquartile range 24–60). The median injected dose was 20 mCi (range 10–25), and PYP was used by 90% of sites in this sample. Planar imaging with single photon emission computed tomography (SPECT) reconstruction was performed at all sites, including seven sites that performed SPECT/CT reconstruction. Lastly, only 50% of sites relied on evidence of myocardial uptake by SPECT to confirm the diagnosis of ATTR cardiomyopathy, while the rest relied on visual assessment and heart/contralateral ratio.

**Conclusion:**

This study is the first to describe variation in imaging practices across sites in the MENA region, especially in acquisition protocols and interpretation standards. Eliminating heterogeneities identified by this study will harmonize image interpretation and reporting and will facilitate successful conduct of regional multi-centre studies.

## Graphical Abstract

**Graphical abstract:**
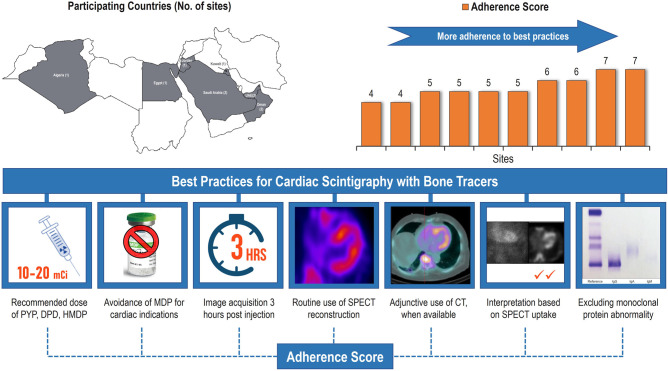
Adherence to imaging best practices at sites participating in PYP-MENA study.

## Introduction

Cardiac scintigraphy with ^99m^Tc-labelled bone-seeking tracers is currently considered the gold standard for non-biopsy diagnosis of transthyretin cardiomyopathy (ATTR-CM).^1,2^ Several professional societies in North America and Europe have emphasized selection of appropriate patients for testing and provided guidance on best practices to improve the quality and yield of radionuclide imaging for diagnosis of ATTR-CM.^2–6^ A key objective of these guidelines is decreasing the number of false positive studies secondary to blood pool activity through greater emphasis on routine incorporation of single photon emission computed tomography (SPECT) reconstruction and relying on SPECT myocardial tracer uptake for study interpretation.^7–9^ Additionally, the guideline strongly endorsed routine testing for monoclonal protein abnormalities to exclude light chain (AL) amyloidosis, especially in cases of mild cardiac uptake of bone-avid tracers, where ATTR-CM may be erroneously diagnosed.^2,5^

However, the extent to which recommended practices have been incorporated at nuclear laboratories around the world remains unknown. Erratic adoption of imaging best practices may be associated with suboptimal study quality at non-adhering laboratories, which conceivably may increase the risk of spurious study interpretations and potentially poor downstream management decisions and patient outcomes.^7,10^ Since non-invasive diagnosis of ATTR-CM is predicated on reliably demonstrating myocardial tracer uptake *in lieu* of performing endomyocardial biopsy, the effectiveness of this paradigm demands consistently performing high-quality radionuclide imaging studies in order to provide unequivocal diagnostic information. As such, widespread incorporation of recommended practices has been encouraged to assure scintigraphy studies predictably meet performance standards, especially with the growing use of non-invasive pathways for ATTR-CM diagnosis beyond major academic centres.^2^

Considering paucity of data on real-world adherence to imaging best practices at laboratories using bone-seeking tracers for cardiac indications, we sought to formally describe adoption patterns for recommended practices at centres in the Middle East/North Africa (MENA) region, where access to radionuclide imaging has been identified as a major obstacle for ATTR-CM diagnosis.^11^ Accordingly, we designed the Appraisal of Amyloidosis Imaging Practices in Middle East and North Africa (PYP-MENA) project to provide needed insight into the current state of amyloidosis imaging in the region, and potentially identify opportunities to optimize utilization and performance of scintigraphy studies at emerging programmes away from amyloidosis centres of excellence in developed countries.

## Methods

### Study design and participating sites

The PYP-MENA is an investigator-initiated, cross-sectional study of sites known to actively utilize bone-avid tracers for cardiac indications in the MENA region. A ‘convenience sample’ of nuclear laboratories in United Arab Emirates, Oman, Bahrain, Kuwait, Saudi Arabia, Jordan, Turkey, Egypt, and Algeria was invited to participate in this study based on personal knowledge of the study investigators that imaging for cardiac amyloidosis was offered in these countries. An online eligibility survey was sent to potential sites to determine their interest and eligibility for participation, and sites with confirmatory responses subsequently received a separate link to the study data collection instrument. Data were reviewed for completeness, and site investigators were contacted for clarifications in case of data inconsistencies or errors.

The study was reviewed by Institutional Review Board and was deemed non-human subject research and hence was exempt from full review and informed consent was waived.

### Data collection and study variables

A data collection instrument was created using an online platform (SurveyMonkey.com). A link to the online instrument was sent via email to the contact person at each eligible site. The instrument included closed-ended questions about site demographics, including location, affiliation, accreditation status, years of operation, types of cardiac imaging systems installed (SPECT, PET, and CT), and types of advanced imaging studies performed at each of these laboratories. In addition, we also collected data on various technical aspects related to the performance and interpretation of cardiac studies involving bone-avid radiotracers, including type of bone-avid radiopharmaceuticals used (PYP, DPD, HMDP, or MDP), injected activity, timing of image acquisition, whether whole body or cardio-centric imaging was performed, criteria routinely implemented for image interpretation (planar vs. heart/contralateral ratio vs. evidence of SPECT uptake), and whether a statement on need to exclude concomitant plasma cell dyscrasia was routinely included in study reports (see Supplementary data online, *Table S1*).

An ‘adherence score’ was calculated for each site based on its adoption of published recommendations on radionuclide imaging for cardiac amyloidosis.^5^ Practices that were incorporated into the calculation of this score included use of recommended radiotracer dose (10–20 mCi of PYP, DPD, or HMDP), avoidance of MDP for cardiac imaging, use of SPECT reconstruction, adjunctive use of CT, routine image acquisition 3-h post-tracer injection, relying on SPECT myocardial uptake for study interpretation, and including a statement on need to exclude plasma cell abnormalities in study report. A score of ‘1’ was assigned for each adopted practice, and the aggregate adherence score assumed values ranging from ‘0’ if a site adhered to none of these practices, to ‘7’ when a site adhered to all practices mentioned.

Descriptive statistics were used to summarize findings, with continuous variables presented as medians and interquartile range (IQR) due to deviation from assumptions of normality, and categorical variables presented as percentages.

## Results

### Site demographics and imaging capabilities

Ten out of 19 invited sites completed the eligibility survey and participated in the study (52.6% response rate). Among participating sites, seven were government-run (70%), one was university-affiliated, and the rest were private laboratories, with 90% of included sites having an active laboratory accreditation status (*Table 1*, Supplementary data online, *Figure S1*). Participating sites have been operating for a median of 15.5 years (IQR 7.75, 23.75) and have been involved with cardiac amyloidosis imaging for a median of 49 months (IQR 24, 60).

**Table 1:** General characteristics of sites participating in PYP-MENA study

Number of sites	10
Country	
Algeria	1
Egypt	1
Jordan	1
Kuwait	1
Oman	2
Saudi Arabia	2
United Arab Emirates	2
Affiliation (%)	
Government-run	8
University-affiliated	1
Private	1
Accreditation	
Yes	8
No	2
Years of operation (IQR)	15.5 (8–24)
Available camera systems (%)^a^	
General purpose SPECT	8 (80%)
SPECT/CT	7 (70%)
Solid-state detector SPECT	1 (10%)
PET or PET/CT	8 (80%)
Advanced imaging services (%)^a^	
SPECT MPI	9 (90%)
PET MPI	4 (40%)
PET (inflammation)	7 (70%)
Coronary CT angiography	7 (70%)
Coronary calcium scan	8 (80%)

^a^Participating sites could have more than one camera system and provide several advanced imaging services. Therefore, total percentages may add up to more than 100%.

Types of installed imaging systems varied among participating sites, with general purpose Anger SPECT cameras available at eight sites, hybrid SPECT/CT systems at seven, PET systems at eight, and solid-state detector SPECT cameras available at only one site. Participating sites offered a wide range of advanced cardiac imaging services as follows: myocardial perfusion with SPECT and PET was performed at nine and four participating sites, respectively, assessment of cardiac metabolism with radioactive-labelled glucose was performed at seven sites, cardiac CT angiography at seven sites, and coronary calcium assessment at eight sites.

### Cardiac scintigraphy with bone-avid tracers

**Figure 1:**
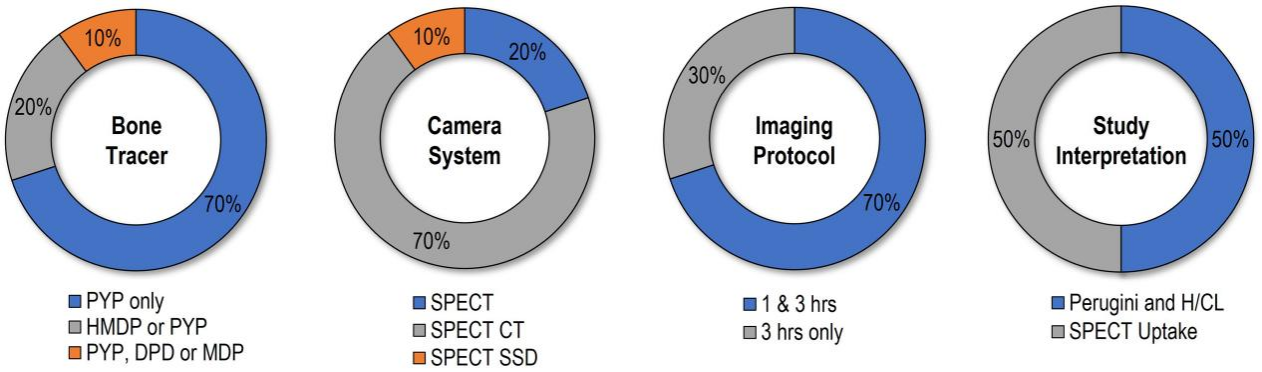
Distribution of site responses to questions on tracers, camera systems, imaging protocols, and interpretation criteria.

Participating sites performed a median of 16 cardiac scintigraphy studies using bone-avid tracers per year (IQR 10, 39). Overall, adherence to practices recommended in recent multi-societal guideline was high among participating sites, with a median adherence score of 5 (IQR 5, 6; range 4–7), and two participating sites adhering to all seven recommended practices (*Graphical abstract*). Median injected radiopharmaceutical activity was 20 mCi (range 10–25), and PYP used exclusively at seven sites, HMDP exclusively at one site, both PYP and HMDP at one site, while one site used PYP, DPD, and MDP. None of the participating sites used planar imaging alone, with 3/10 sites (30%) using planar imaging along with SPECT reconstruction, and the remaining seven used planar imaging with SPECT/CT reconstruction. Image acquisition was performed 1 and 3 h after radiotracer injection by the majority of sites (7/10), while the remaining three sites acquired images only 3-h post-tracer administration. A combination of semi-quantitative tracer uptake assessment on planar images (Perugini grade) and heart to contralateral lung (H/CL) ratio was used to determine whether studies are suggestive of cardiac amyloidosis by 5/10 sites, while the remaining five sites relied on evidence of myocardial tracer uptake by SPECT to identify studies suggestive of cardiac amyloidosis. Lastly, whole body scanning was only performed by two sites, and a statement on need to formally exclude monoclonal protein abnormalities was routinely included in study reports at 4/10 sites (40%), on case-by-case basis at four sites, but never mentioned by the remaining two sites (*Figure 1*).

## Discussion

The Appraisal of Amyloidosis Imaging Practices in the Middle East/North Africa (PYP-MENA) study provides the first formal assessment of real-world adoption of imaging best practices with cardiac scintigraphy using bone-avid tracers in a sample of nuclear laboratories in the MENA region. The current data demonstrate high levels of adherence to contemporary guidelines, including use of recommended tracers and dosages, and performance of SPECT reconstruction (with or without CT) by almost all sites in this sample. In addition to providing needed reassurance, our study also identifies certain gaps primarily related to imaging protocols and study interpretation.

A striking finding of the current study is that half of included sites relied on semi-quantitative visual assessment and H/CL ratio for study interpretation, but not evidence of myocardial tracer uptake by SPECT, despite availability of SPECT at all laboratories in this sample. Semi-quantitative grading and H/CL ratio determination have been deemed insufficient for study interpretation in the updated multi-societal guideline,^5,6^ while demonstration of diffuse or focal myocardial uptake on SPECT was shown to decrease the incidence of false positive studies and was therefore emphasized as an essential step in systematic study interpretation.^8,9,12^ Reasons behind overreliance on planar imaging-based parameters for study interpretation and whether it has adversely impacted diagnostic yield or downstream resource utilization at these laboratories remain unknown. Relying exclusively on planar imaging for study interpretation may arguably lead to a higher incidence of false positive studies, which can lead to a presumed diagnosis of ATTR-CM. This may potentially have serious consequences, including incorrectly labelling a patient with an erroneous diagnosis, initiating unnecessary and expensive medications, and most importantly, missing or delaying the diagnosis of AL amyloidosis. As such, adherence to published guidelines for interpretation of cardiac scintigraphy studies involving bone-seeking tracers can potentially have significant patient-level and societal benefits. Moreover, updated criteria emphasizing the importance of detecting myocardial tracer uptake with SPECT should be unequivocally mentioned by all professional societies producing consensus guideline documents to eliminate any potential confusion.^6^

An additional relevant observation of this study is the variation in the timing of image acquisition post-tracer injection. More than two-thirds of participating sites performed imaging at both 1 and 3 h post-injection, despite current recommendations considering 1-h acquisition optional, regardless of injected tracer.^5^ These findings, however, reflect the prevailing debate in current literature on best timing for image acquisition, with several studies indicating that 1-h-only protocols might be efficiently performed without compromising diagnostic yield of the test, whereas the consensus guideline requires image acquisition 2.5–3-h post-tracer injection.^5,6,8,13,14^ While we are unable to speculate if participating sites adopted these practices based on accumulated experience, unique workflow requirements, or other reasons, our findings underscore the need to eliminate such protocol heterogeneities and to harmonize imaging practices across laboratories in the region. Standardization of imaging and reporting will likely facilitate conduct of future regionwide studies aiming to assess real-world utility of bone tracer cardiac scintigraphy for diagnosis of amyloidosis.

In this sample, less than half of participating sites routinely reported a statement on importance of excluding plasma cell abnormalities in study reports, and an additional 40% included such a statement on a case-by-case basis. Given potential grave consequences that might be associated with missed or delayed diagnosis of AL amyloidosis,^15^ including a clear recommendation to perform monoclonal protein evaluation is now considered a required element of study reports.^5^ Contemporary approach to exclude plasma cell abnormalities involves the use of serum free light chain assays along with serum and urine immunofixation.^16^ Routine inclusion of a statement on appropriate next steps to exclude AL amyloidosis provides referring physicians with needed guidance, especially since common practice patterns in the MENA region may not fully align with existing literature.^11^

### Limitations

We acknowledge that the ‘convenience sample’ of nuclear laboratories in this study may not be representative of the entire MENA region, and that the high reported levels of adherence may not reflect prevailing practice patterns at unrepresented sites or countries. In fact, we postulate that adherence to imaging best practices at non-represented sites in the region may actually be lower, since imaging centres that participated in this study are likely experienced, as evidenced by availability of advanced imaging capabilities and the number of years these laboratories have been operational. Secondly, data on imaging findings and number of diagnosed ATTR-CM cases at each site are lacking, which precludes examining the association between site adherence scores and diagnostic yield of scintigraphy studies, downstream resource use, or patient outcomes. Lastly, we were unable to capture temporal changes in adoption of best practices or how cardiac amyloidosis imaging evolved at participating sites due to the cross-sectional nature of the study.

Despite these limitations, the current study has notable strengths, including broad representation of countries in the MENA region and utilization of a detailed data collection tool that allowed capture of granular data on various technical aspects of test performance. Moreover, the current study provides a feasible framework that can be adopted in future larger scale initiatives across the region, with the aim of generating more generalizable knowledge and identifying opportunities to improve imaging practices in the MENA region. In addition, the role of emerging imaging modalities in diagnosing and differentiating various cardiac amyloidosis phenotypes, including positron emission tomography, can be examined in multi-centre studies using the framework tested in PYP-MEAN. Taken together, the PYP-MENA findings will inform development of quality improvement initiatives designed to advance the field of amyloidosis imaging in the region.

## Conclusion

In the first formal assessment of cardiac amyloidosis imaging practices in the MENA region, we report high levels of adherence to recommended best practices at a sample of experienced imaging centres in the region. However, we also report several gaps, particularly in relation to lack of standardization of image acquisition and study interpretation. Our findings provide unique insight into the practice of advanced cardiac imaging in the MENA region, which will guide regional quality improvement initiatives aiming at optimizing test performance and reporting. Furthermore, the current study framework demonstrates the feasibility of future multi-centre studies in the region to examine real-world utility of cardiac scintigraphy with bone-seeking tracers.

## Supplementary data

Supplementary data is available at *European Heart Journal - Imaging Methods and Practice* online.

## Supplementary Material

qyad025_Supplementary_Data

## Data Availability

Underlying data will be shared on reasonable requests to the corresponding author.

## Lead author biography

Dr. Firas Al Badarin is a consultant cardiologist and outcomes researcher at the Heart and Vascular Institute in Cleveland Clinic Abu Dhabi. He is passionate about judicious use of advanced imaging modalities in patients with various cardiovascular illnesses, especially cardiac amyloidosis.
