# Supplementary material for: Appraisal of amyloidosis imaging practices in the Middle East/North Africa (PYP-MENA)
Source: Eur Heart J Imaging Methods Pract. 2024 Jan 16;2(1):qyad025. doi: 10.1093/ehjimp/qyad025 (PMC11195776; doi:10.1093/ehjimp/qyad025)
Supplement: qyad025_Supplementary_Data [file qyad025_Supplementary_Data.zip › Supplementary Table S1.docx]

**Supplementary Table S1:** Appraisal of Amyloidosis Imaging Practices in the Middle East/ North Africa (PYP-MENA) data collection tool

| 1. **Name and location of nuclear medicine/ nuclear cardiology laboratory** | | | | | | | | | | | | | | | | |
| --- | --- | --- | --- | --- | --- | --- | --- | --- | --- | --- | --- | --- | --- | --- | --- | --- |
|  | | | | | | | | | | | | | | | | |
| 1. **How can the affiliation of your laboratory be best described?** | | | | | | | | | | | | | | | | |
| - Private facility | | | | | - Government-run | | | | | | | | - University-affiliated | | | |
|  | | | | | | | | | | | | | | | | |
| 1. **How can the accreditation status of your laboratory be best described?** | | | | | | | | | | | | | | | | |
| - ARC accredited | | | - IAC accredited | | | | | | - Other accreditation | | | | | - Not accredited | | |
|  | | | | | | | | | | | | | | | | |
| 1. **Number of years this laboratory has been operational:** | | | | | | | | | | | | | | | | |
|  | | | | | | | | | | | | | | | | |
| 1. **What kind of imaging equipment is used for cardiac indications in your laboratory (check all that applies)?** | | | | | | | | | | | | | | | | |
| - Anger cameras | | | - SPECT/ CT | | | | | | - PET/ CT | | | | | - Solid-state detector | | |
|  | | | | | | | | | | | | | | | | |
| 1. **Which of the following cardiac studies is performed at this laboratory (check all that applies)?** | | | | | | | | | | | | | | | | |
| - SPECT MPI | - PET MPI | | | | | - PET FDG | | | | - Cardiac CTA | | | | | | - Ca scan |
|  | | | | | | | | | | | | | | | | |
| 1. **Number of years cardiac scintigraphy for cardiac amyloidosis has been performed at this site:** | | | | | | | | | | | | | | | | |
|  | | | | | | | | | | | | | | | | |
| 1. **Number of scintigraphy studies performed in the last year for cardiac amyloidosis:** | | | | | | | | | | | | | | | | |
|  | | | | | | | | | | | | | | | | |
| 1. **Which of the following radiotracers is used for cardiac imaging at your laboratory (check all that applies)?** | | | | | | | | | | | | | | | | |
| - PYP | | | - DPD | | | | | | - HMDP | | | | | - MDP | | |
|  | | | | | | | | | | | | | | | | |
| 1. **What radiotracer dose is most commonly used for these studies (mCi)?** | | | | | | | | | | | | | | | | |
| - 10 | | - 15 | | | | | - 20 | | | | - 25 | | | | - >25 | |
|  | | | | | | | | | | | | | | | | |
| 1. **Which technique is used for image acquisition of cardiac scintigraphy studies with bone seeking tracers?** | | | | | | | | | | | | | | | | |
| - Planar imaging only | | | | | - Planar + SPECT | | | | | | | | - Planar + SPECT/ CT | | | |
|  | | | | | | | | | | | | | | | | |
| 1. **Which camera type is used for performance of cardiac scintigraphy studies with bone seeking tracers?** | | | | | | | | | | | | | | | | |
| - General purpose SPECT | | | | | - SPECT/ CT | | | | | | | | - Solid state detector SPECT | | | |
|  | | | | | | | | | | | | | | | | |
| 1. **At what time intervals is image acquisition performed for cardiac scintigraphy studies with bone seeking tracers?** | | | | | | | | | | | | | | | | |
| - 1-hour only | | | | | - 3-hour only | | | | | | | | - Both 1&3 hours | | | |
|  | | | | | | | | | | | | | | | | |
| **14. Which of the following criteria is used to determine whether a study is strongly suggestive of cardiac amyloidosis?** | | | | | | | | | | | | | | | | |
| - Perugini score alone | | | - H/ CL alone | | | | | | - Perugini + H/ CL | | | | | - Uptake on SPECT | | |
|  | | | | | | | | | | | | | | | | |
| **15. Is whole body scanning performed routinely at your laboratory?** | | | | | | | | | | | | | | | | |
| - Yes | | | | | | | | - No | | | | | | | | |
|  | | | | | | | | | | | | | | | | |
| **16. Is a statement on need to exclude monoclonal protein abnormality is routinely included in your reports?** | | | | | | | | | | | | | | | | |
| - Yes | | | | - No | | | | | | | | - It depends | | | | |
