# Supplementary figures and images for: Appraisal of amyloidosis imaging practices in the Middle East/North Africa (PYP-MENA)
Source: Eur Heart J Imaging Methods Pract. 2024 Jan 16;2(1):qyad025. doi: 10.1093/ehjimp/qyad025 (PMC11195776; doi:10.1093/ehjimp/qyad025)

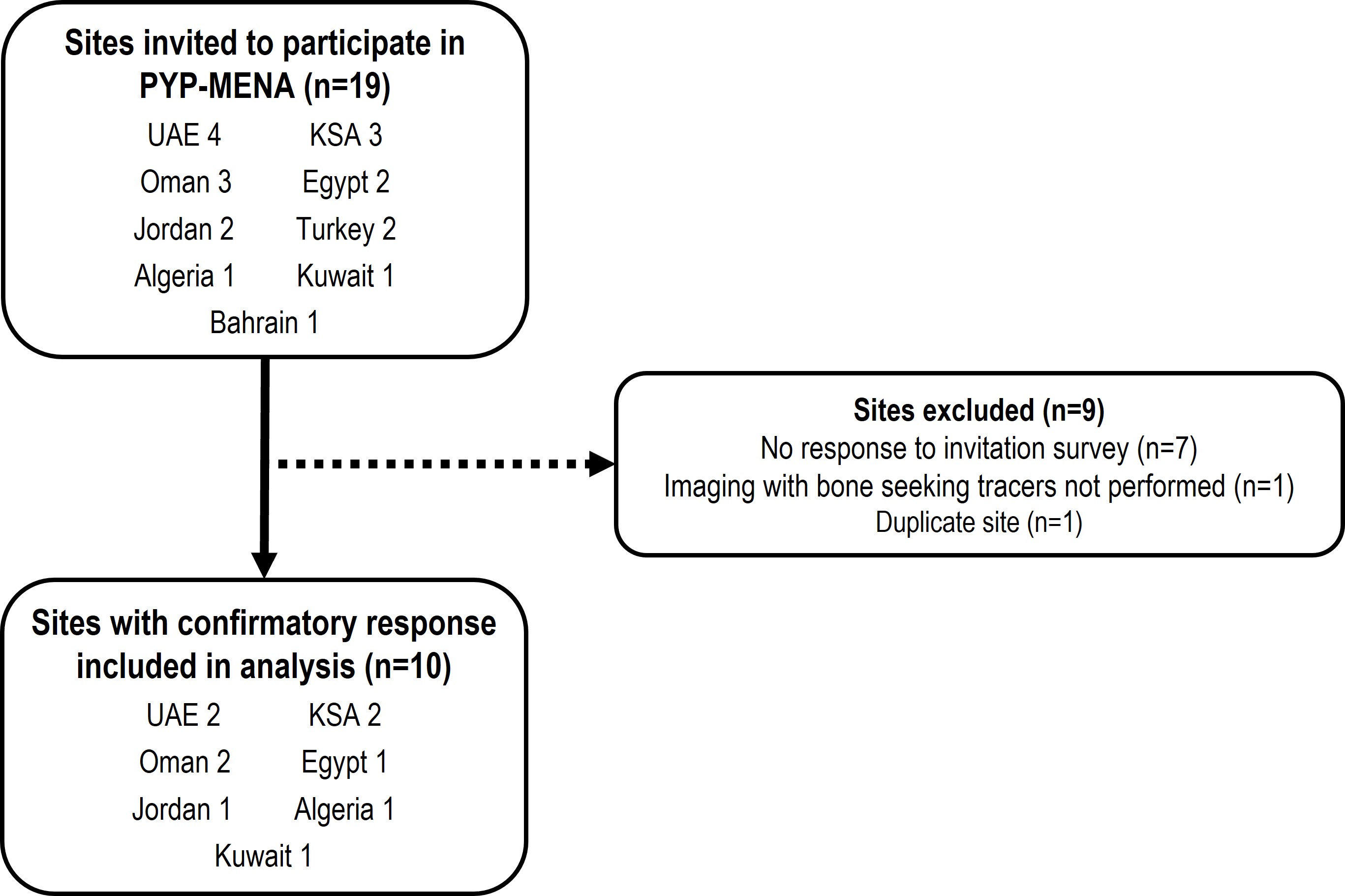

Supplement: qyad025_Supplementary_Data [file qyad025_Supplementary_Data.zip › Figure S1.jpg]
